# The Nervous Patient

**Published:** 1889-05

**Authors:** J. R. Callahan

**Affiliations:** Hillsboro, O.


ARTICLE V.

### THE NERVOUS PATIENT.

BY J. R. CALLAHAN, D. D. S., HILLSBORO, O.

[Read before the Mississippi Valley Dental Society, Cincinnati, March 7th, 1889.

Who of the dental profession does not dread to come in contact with the nervous patient, or perhaps we might say the patient of hyper-nervous temperament, who, by his hysterical conduct in the chair seems to sap the operator of all the vitality he ever possessed, and make him feel as if palsied old age had come upon him, and finally when the patient is gone exclaim, "I'm glad that patient is gone; I really declare the patience of Job would yield at the chair." Be the patient a lady, she will, perhaps, begin by telling you how she dreads the awful boring machine, the horrid rubber dam, etc., etc. She will get into your chair with wailings and moanings; the moment you begin to operate, she will begin to jump, perhaps grab your hands, declaring she can not possibly permit you to proceed. If the nervous patient be a big strong man he will get into the chair with many misgivings, and after one or two futile efforts to stand the operation, conclude you had better pull the—tooth out.

This pain, and dread and nervousness, is a reality with most of our patients, especially so to those of nervous temperament, and I am of the opinion that most of the fault lies with the dentist. Hundreds of people are needlessly tortured every day by the dentists of the land. This is all wrong; there is no necessity of intense suffering in the dental chair. The dentist inflicts needless punishment generally for one of three reasons, viz: parsimoniousness, carelessness, or ignorance. Parsimonious in that he will insist on using dull and worn out instruments, is too stingy to subscribe for journals that he may keep posted and keep his mind active in most modern theories and practices of his profession. Careless in regard to condition of instruments; careless as to the amount of pain inflicted; careless as to reputation; seemingly unmindful of everything only to get through and get the fee. Ignorance either from lack of appliance, or lack of ability to learn of, and apply the many agents and methods of lessening pain in dental operations.

Local pain obtundents are offered without number, a few good, but a large majority of them worse than useless; especially useless are the patent obtundents.

It has been stated that by thoroughly drying cavities with alcohol and warm air and excavating with sharp instruments, all that is necessary for the obtunding of sensitive dentine and allaying accompanying nervousness has been done.

This has not been my experience; while thoroughly drying cavities and using sharp instruments will go a great way toward comfortable operations, yet, there are many cavities on which this method seems to have but little of the desired effect.

Dr. F. H. Brimmer, in the *Independent Practitioner*, vol. 8, page 301, advocates a very unique pain obtunder. He claims to be able to obtund the tooth pulp by vibrations caused by a rapidly revolving instrument. He cut off the crown of a central incisor containing a living pulp in the following manner: "A large spear-shaped drill was held in light contact with the centre of the tooth at the gum line, and revolved rapidly for fifteen or twenty seconds, the contact being only sufficient to transmit vibrations. The drill was then pressed against the tooth with a little more force, and the sensibility being diminished, it was allowed to penetrate perhaps one-third the distance to the pulp, when it was again used as at first. At the third trial the drill entered the pulp; with a probe, carbolic acid crystals were gradually forced to the end of the root; in twenty-three minutes the tooth was cut off and properly shaped for the crown which was set on the following day. The whole was accomplished almost without pain." He goes on to give the following theory in regard to the operation. He says: "There is a limit to nerve conductibility. If a nerve be continuously irritated, after a time it loses its power of transmission until time has been given for it to recover its normal condition. The continual passing of the drill exhausts the irritability of the nerve, and it no longer conveys sensation until it has had time to recover its tone." I have not as yet tried this, to me, novel obtundent, but from what I know of Dr. B., he means just what he says; and I think the method worth a trial.

Local obtundents proving themselves so very unsatisfactory many practitioners are administering chloroform for dental operations. This is a practice that can not be too strongly condemned. Many dentists administer this very dangerous drug while excavating sensitive teeth; they will tell you that they only carry the patient to the first stage, thereby avoiding danger. This, as I understand authorities on chloroform, is perhaps the most dangerous stage for dental operations. It is stated in various places by authorities that especial danger depends on the well known fact that any operation which involves irritation to the sensory terminations of the fifth pair of nerves excites the inhibitory action of the pneumogastric nerves (through their sympathetic connections), which causes slowing of the heart's action and may terminate in fatal syncope. In the light of these and many other facts that might be mentioned, would it not be proper to say that the use of chloroform in dental operations is criminal practice ?

In the *Dental Cosmos*, vol. 27, page 12, Dr. W. H. Dwinelle, of New York, gives a very interesting experience in the use of morphine and atropine for the purpose of quieting very nervous patients. He used the morphine for its anodyne qualities and the atropine for the purpose of overcoming the objectionable qualities of the morphine, and for the further purpose of checking the flow of the saliva. His manner of introducing it into the system is as follows: Start with one-sixth grain of atropine combined with one-eighth grain of morphine—dose repeated in an hour; increase dose of morphine if found necessary. Dr. Dwinelle and many other prominent dentists report very satisfactory results from this treatment. I would advise all who have not already done so, to read carefully this article referred to. This treatment like any other should receive careful attention before adopting or using it at all. I had a very agreeable experience within the short time I used it. I abandoned the practice on account of the time it consumed. In casting about for means to effectually and safely quiet the disagreeable nervousness of many patients, without taking up so much time, I began to try nitrous oxide gas. After having used it more or less for about four years I have adopted the following method: After having opened the cavity with a chisel, get instruments I wish to use all ready; dry cavity with an absorbent; administer from three to six inhalations of gas; this, as you know, is a very small dose or almost no dose at all, yet it is sufficient to have a very quieting and delightful effect upon the nervous system. The patient does not lose consciousness but seems to lose that nervous dread or apprehension so often described by saying that the tooth felt as if the instrument was going clear through to the pulp. When asked if they felt any pain the patient will usually say no; or perhaps it may be as a lady said to me a few days since, when I inquired if she felt any pain, she replied, "Yes, a little, but I felt so calm and restful that I did not mind it in the least."

The gas should not be given in sufficient quantities to produce anæsthesia, but simply enter the first stage, called by Professor Guilford, the exhilarating symptom. Two administrations as described will enable the operator to prepare the sensitive portion of any ordinary cavity. Many people refuse to take it, being afraid of the gas. I never insist on it except in the case of very nervous persons. If there be any danger in the use of nitrous oxide gas as here proposed, I have been unable to find it, either from the history of the anæsthetic or from practice; if there is any let us hope it may be made apparent in the discussion of the demerits of this rather disconnected and incomplete paper.—*Dental Register*.

